# Ag-CeO_2_ Based on Electrochemical Sensor for High-Efficient On-Site Detection of Nitrite in Aquaculture Water and Beverages

**DOI:** 10.3390/molecules29112644

**Published:** 2024-06-04

**Authors:** Kunmeng Zhao, Ziyao Zhang, Yihui Zhou, Xuexia Lin

**Affiliations:** 1 College of Chemical Engineering, Huaqiao University, Xiamen 361021, China; 2 College of Materials Science and Engineering, Huaqiao University, Xiamen 361021, China

**Keywords:** Ag-CeO_2_, nitrite, electrochemical sensor, beverage testing, aquaculture water

## Abstract

Nitrite is one of the most common nitrogenous compounds, which is not only an important indicator of aquaculture water but also widely used as a food additive. Its potential toxicity poses a huge threat to aquatic products and human health. Therefore, it is important to develop a convenient and rapid sensor for the high-efficient onsite detection of nitrite. In this work, a novel electrochemical sensor was developed for the qualitative and quantitative analysis of nitrite. The developed nitrite electrochemical detection system is easily applied in onsite detection. The electrochemical working electrode was constructed based on the combination of Ag-CeO_2_ and conductive carbon paste (CPE) with excellent electrocatalysis activity and rapid electron transfer ability. By the application of the developed system and under the optimal conditions, the linear range was from 40.0 μM to 500.0 μM, and the detection limit was reduced to 4.3 μM. The recovery was between 92.1% and 108.1%, and the relative standard deviations (RSDs) were 0.49%~9.31%. The sensor exhibited superior reproducibility, high stability sensitivity, and anti-interference ability, confirming its effectiveness for nitrite analysis. Finally, the developed electrochemical sensor was successfully applied to detect nitrite in beverages and aquaculture water samples, indicating that this approach has great potential in onsite food testing and environmental monitoring.

## 1. Introduction

Nitrite is one of the most common nitrogen-containing compounds in the aquaculture water environment, serving as a critical indicator for water quality monitoring [1]. Additionally, nitrite is widely used as a kind of food additive to enhance food coloring and preservation [2]. However, small amounts of inhalation and a long-term intake can lead to severe diseases and even death in aquatic products and can cause acute poisoning and cancer in humans, posing a significant threat to human health [3]. Moreover, there is also a threat to human life with the long-term intake of excessive nitrite in the daily dietary routine. For example, overdosing with nitrite will do damage to the digestive system and cause cardiovascular disease. In addition, nitrite can react with amines in the human body to generate nitrosamines, which are highly carcinogenic, and this substance is especially easily formed in the acidic environment of the stomach, increasing the risk of intestinal and gastric cancer [4]. Due to the high hazards of nitrite for humans, nitrite has been classified as a carcinogen by The World Health Organization, and many countries have implemented laws and regulations to restrict and control its use in food. Therefore, substantial efforts have been directed towards developing high-efficient sensors to monitor and control nitrite levels.

In recent years, colorimetry sensors [5], fluorescence sensors [6], surface-enhanced Raman scattering sensors [7], microfluidic sensors [8,9], and electrochemical sensors have been extensively developed for rapid onsite analysis and portability [10]. Colorimetry sensors and spectroscopy sensors, including UV–vis spectroscopy and fluorescence spectroscopy, often have interference from substrates in samples and non-specific adsorption, limiting their accuracy in nitrite analysis. Although the methods for nitrite detection, based on chromatography, such as high-performance liquid chromatography (HPLC) or capillary electrophoresis, possess high precision [11], they are overly burdensome, generally tedious, and expensive for routine analysis. Given the limitations of these methods, electrochemical sensors present an attractive alternative due to their ease of operation, low cost, rapid analysis, high portability, and ease of integration. Consequently, an electrochemical sensor is considered as one of the most powerful tools for nitrite analysis. It is well known that the sensing mechanism is based on the electrooxidation of nitrite, where the rate of oxidation reaction depends on the electron transfer kinetics and electroactive ability of electrode materials. Therefore, a rapid and simple nitrite detection sensor can be achieved by utilizing suitable electrode modification materials.

During the past decade, materials such as glassy carbon (GC), platinum, silver materials [12], gold materials, titanium [13], copper materials, and cerium (Ce) nanomaterials have been commonly used for electrode modification materials [14]. The researcher Lu [15] designed and synthesized a novel and stable nanoporous Pt/CoO via Al_85_Co_14_Pt_1_ ternary alloy, which exhibits high electrocatalytic activity due to the Pt nanoclusters doped within the CoO crystal structure. The detection of nitrite in the range of 0.2 μM–3.67 mM and 3.67–23.7 mM was realized. Zhang et al. [16] synthesized Dy_2_(WO_4_)_3_ by a hydrothermal method and modified gold nanoparticles on its surface, and the sensor showed excellent electrocatalytic activity, stability, reproducibility, and selectivity during electrooxidation in the presence of gold, which can be used for the determination of NO_2_^−^ in human urine samples. In addition, Chen et al. [6], utilized the binding affinity of gold nanoparticles (AuNPs) to the sulfhydryl or amino groups on the surface of methanobacteria (Mb), and realized the fluorescence sensing of nitrite in the low concentration ranges of 0–8.0 μM and 8.0–50.0 μM, This was based on the effect of the diazotization-coupling reaction of Mb with nitrite on the fluorescence of AuNPs.

Cerium dioxide (CeO_2_) nanomaterials are one of the most common redox materials [14]. The Ce^4+^ ions in CeO_2_ can be easily converted into stable Ce^3+^, releasing reactive oxygen species to maintain the charge balance. Manibalan et al. [17] applied heterostructured CeO_2_ materials-based doped NiO nanocomposites as electrode materials, achieving higher electrochemical performance, energy density, selectivity, and stability, compared to pristine CeO_2_@NiO. Rudayni et al. [18] synthesized CeO_2_ nanoparticles via a hydrothermal method for the electrochemical detection of furantoin (NFT) antibiotics. The electrodes based on these nanomaterials exhibited improved electrocatalytic activity for the oxidation of NFT, with a limit of detection (LOD) of 7.81 μM. However, challenges such as poor stability remain. To address the inherent limitations of CeO_2_ in electrochemical sensors, it is often doped with other metal nanomaterials that possess high electrical conductivity and stability. The slow electron transfer kinetics of CeO_2_ can be enhanced through doping or adding metals or metal oxides, resulting in a synergistic effect between the active sites of CeO_2_ and noble metals. The metal/CeO_2_ composites have been extensively studied in oxidation reactions, such as Pd [19,20], Pt [15,21], Ru [22], Au [16,23], and Ag matrix composites [24]. Although both Pt and Au nanomaterials can improve the catalytic activity and achieve the highly sensitive detection of nitrite, their high cost and rarity limit their further development in nitrite detection applications. Ag, as a material with high electrical conductivity, high catalytic activity, a reasonable price, and easy accessibility, has become a hot spot for the electrochemical detection of nitrite in recent years [25]. The interaction between the Ag^+^/Ag^0^ and Ce^3+^/Ce^4+^ pair is facilitated by Ag particles and CeO_2_ oxygen species [26], enhancing the electrochemical activity and stability of Ag-CeO_2_ composites. However, numerous studies have found that metals, whether pure or doped, are prone to contamination due to their active redox properties, which compromise the stability of detection. For instance, Nouri et al. developed a novel electrochemical biosensor for the highly sensitive detection of rizatriptan benzoate in pharmaceutical preparations and biological samples by modifying a carbon paste electrode (CPE) with double-stranded DNA, nickel ferrite magnetic nanoparticles (NiFe_2_O_4_NPs), and gold nanoparticles (AuNPs), and the sensor exhibited high selectivity and stability [27]. Nithyayini’s team developed a sensing platform integrating NiFe_2_O_4_ nanoparticles into a CPE, which significantly enhanced the nitrite monitoring sensitivity in the range of 0.1 to 1000 μM due to the combination of the nanomaterials and conductive carbon, which resulted in the enhanced catalytic activity and stability of the sensor in oxidizing nitrite [28]. CPE is frequently utilized as a modifying material for electrochemical sensors because of its low price and high stability. Pontie et al. [29] developed a novel electrode material combining cetyltrimethylammonium bromide (CTAB) with CPE for analyzing nitrite in aquaculture and rat blood. With the addition of CPE, the repeatability and reproducibility of the electrode improved to varying degrees, and the electrode had good ion selectivity, offering the possibility of blood testing with complex compositions. Based on these findings, there is a strong rationale to hypothesize the development of a convenient and straightforward electrochemical nitrite detection sensor that integrates Ag-CeO_2_ nanomaterials and CPE. This combination is expected to catalyze the rapid conversion of nitrite to nitrate, while also enhancing sensor stability.

**Figure 1:**
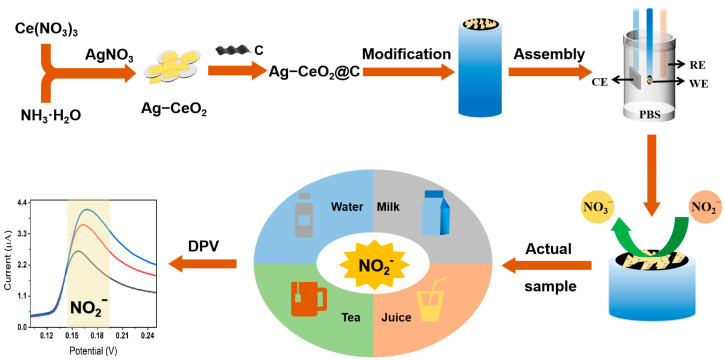
Ag-CeO_2_ electrochemical sensor for the detection of nitrite.

To validate the hypothesis, a simple and convenient electrochemical sensor for rapid nitrite assay was developed, utilizing Ag-CeO_2_ nanomaterials, as illustrated Figure 1. To increase the interfacial active sites and oxygen vacancies, Ag-CeO_2_ nanomaterials were doped with conductive carbon paste (CPE), forming the composite Ag-CeO_2_@C. This composite was employed as an electrode modification material to improve the sensitivity, response time, and stability. Due to its portability, onsite assays were readily accomplished with the developed sensor. The developed method was validated in terms of the linearity, sensitivity, repeatability, and accuracy. Ultimately, the sensor was applied to detect nitrite in aquaculture water and beverages. Thus, we believe this simple, convenient, and portable electrochemical sensor is useful for nitrite assay and has the potential to serve as a reliable onsite detection method in aquaculture and the food industry.

## 2. Results and Discussion

### 2.1. Characterization of Ag-CeO_2_ Nanoparticles

**Figure 2:**
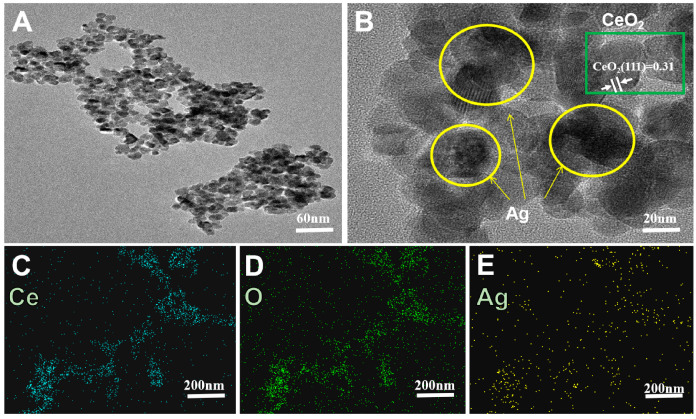
(**A**) Low magnification TEM images of Ag-CeO_2_. (**B**) High magnification TEM images of Ag-CeO_2_. (**C**–**E**) Elemental analysis images of Ce, O, and Ag.

For the construction of a rapid response electrode sensor, the Ag-CeO_2_ nanoparticles were prepared. The morphology and microstructure of Ag-CeO_2_ nanoparticle were characterized by HRTEM technology. Ag-CeO_2_ nanoparticle were observed with an approximate size of 10 nm. It can be seen that Ag nanoparticles were attached to the surface of CeO_2_ nanoparticles. As shown in Figure 2B, the (111) lattice plane of CeO_2_ was identified with a spacing of 0.31 [30], confirming the successful preparation of Ag-CeO_2_ nanoparticles, as depicted in Figure 2A,B. Moreover, the EDS elemental mapping analysis also showed that the nanoparticles are composed of Ag, Ce, and O, as in Figure 2C–E. The distribution of the Ag is regularly around the Ce and O, further verifying that the Ag-CeO_2_ nanohybrid was successfully synthesized.

**Figure 3:**
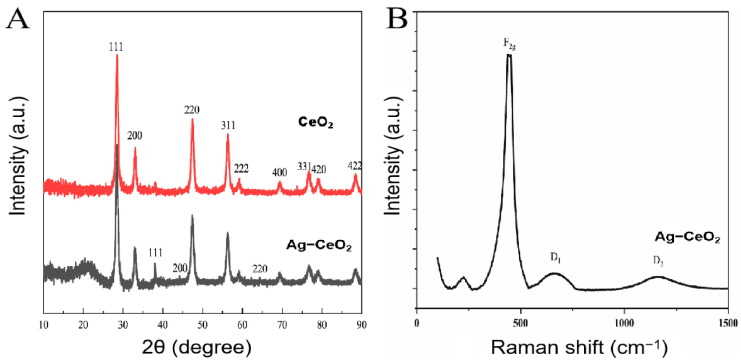
(**A**) XRD spectrum of CeO_2_ and Ag-CeO_2_. (**B**) Raman spectra of Ag−CeO_2_.

XRD patterns were performed on the CeO_2_ and Ag-CeO_2_ nanoparticles, as shown in Figure 2A. It can be seen that the CeO_2_ has a classical crystalline fluorite structure with multiple characteristic diffraction peaks. Diffraction peaks of the CeO_2_ with 2θ = 28.5°, 33.1°, 47.5°, 56.3°, 59.1°, 69.4°, 76.7°, 79.1°, and 88.4° correspond to the (111), (200), (220), (311), (222), (400), (331), (420), and (422) planes, respectively (JCPDS card No. 34-0394) [31]. It was found that the face-centered cubic structure of the Ag nanoparticles can be observed at 2θ = 38.2°, 44.2°, and 64.8° corresponding to the (111), (200), and (220) planes [32]. The fact is that there are structural defects in the Ag-CeO_2_ nanoparticles because silver nanoparticles coexist in the lattice of cerium atoms and due to the structural isomerization of Ag-CeO_2_ itself [30]. The XRD results confirm that Ag nanoparticles were successfully loaded on the CeO_2_ surface. To further understand the structure of the composites, Raman spectroscopy was also carried out, as shown in Figure 3B. The sample shows a high intensity Raman peak near 454 cm^−1^, which is due to the typical F_2g_ vibrational mode of CeO_2_ [33]. In addition, the Ag-CeO_2_ nanomaterials have a broad peak in the 520–760 cm^−1^ region, which corresponds to the oxygen vacancies in CeO_2_ that arise from Ag doping into the cerium lattice. This region also corresponds to the defect-induced mode D_1_ [34], which is consistent with the XRD results. In addition, the D_2_ Raman peak in the 1100 cm^−1^ region is related to the high content of the oxygen vacancies on the surface of Ag-CeO_2_, which provides sufficient active sites for the reaction of nitrite oxidation.

### 2.2. Fabrication of Ag-CeO_2_ Based on an Electrochemical Sensor

**Figure 4:**
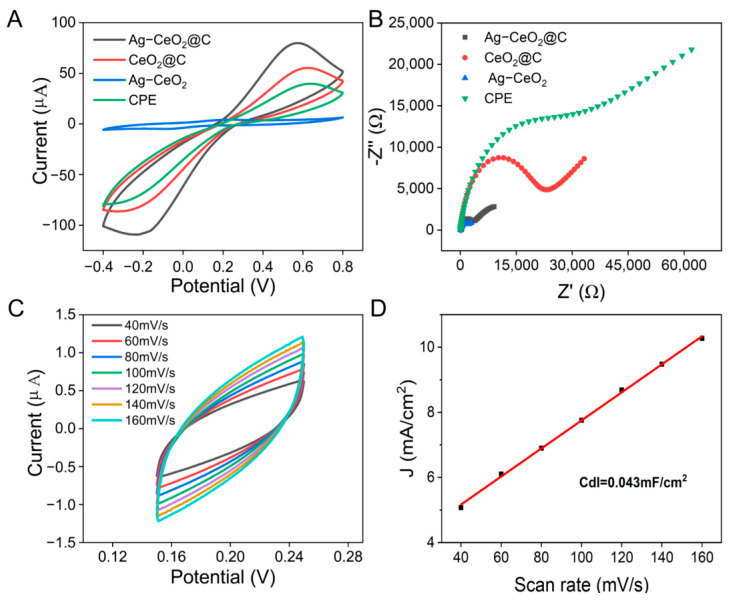
(**A**) CVs of the CPE, Ag-CeO_2_, CeO_2_@C, and Ag-CeO_2_@C in 5 mM K_3_[Fe(CN)_6_] and 0.1 M KCl at a scan rate of 100 mV s^−1^. (**B**) EIS test. (**C**) CV test at different scanning speeds. (**D**) Linearity between the current and scan rate for ECSA testing.

The feasibility of the prepared Ag-CeO_2_@C electrochemical sensor was firstly investigated by CV measurements and electrochemical impedance spectroscopy (EIS). A 50 mL 0.1 M KCl solution containing 5 mM K_4_Fe(CN)_6_/K_3_Fe(CN)_6_ was used to assess the electrochemical behavior. Figure 4A shows the CV curves of CPE, Ag-CeO_2,_ CeO_2_@C, and Ag-CeO_2_@C, respectively. A pair of reversible redox peaks can be observed, and the initial potentials of the four electrodes are 0.63 V, 0.20 V, 0.61 V, and 0.57 V, respectively. The redox potentials of Ag-CeO_2_ are −0.102 V and 0.197 V, respectively. Among them, the low redox potential difference of Ag-CeO_2_ indicated the fastest electron transfer ability. The change in the initial potentials of Ag-CeO_2_@C demonstrated that Ag-CeO_2_ in CPE has the largest ability to accelerate electron transfer. When CeO_2_@C was applied, the intensity of the reversible redox peak was increased, demonstrating that the CeO_2_ nanomaterials provide additional oxygen vacancies and active sites. When the Ag-CeO_2_@C composites were modified at the surface of the GCE, a significant enhancement in the reversible redox peak signals was observed, demonstrating the synergistic effect of Ag-CeO_2_ on enhancing electron transfer and catalytic activity. The higher peak currents and faster electron transfer of the sensors might be related to the increased electrical conductivity and catalytic activity. The EIS was then studied. The high-frequency semicircle part in the EIS corresponded to the electron transfer-limited process, and its diameter was equal to the electron transfer resistance of the electrode. As shown in Figure 4B, the semicircle of CPE was the largest, and the corresponding charge transfer resistance was 32.06 KΩ, while the radius of the semicircle decreased with the addition of CeO_2_, and the charge transfer resistance became 23.34 KΩ. The charge transfer resistance of Ag-CeO_2_@CPE was only 3.49 KΩ, which suggested that the addition of Ag-CeO_2_ nanoparticles can improve the electrical conductivity and catalytic performance of the sensor.

In order to understand the electrochemical active specific surface area of the prepared sensor, the electrochemical active surface area (ECSA) of the sensors was investigated by calculating the bilayer capacitance (Cdl) from the static CV curves in the absence of Faraday reaction processes. According to the equation ECSA = Cdl/Cs [35], the Cdl of Ag-CeO_2_@C was 0.043 mF*cm^−2^, as shown in Figure 4C,D. The value of the specific charge (Cs) in 0.1 M PBS solution at the standard state was 0.02 mF*cm^−2^, and the ECSA of the Ag-CeO_2_@C senor was calculated to be 2.15 cm^2^, compared to just 0.0125 cm² for the CPE sensor. The high ECSA of Ag-CeO_2_@C significantly improved the electrocatalytic ability, which is beneficial for improving the detection rate and sensitivity of nitrite.

### 2.3. Investigation of the Electrochemical Behavior and Mechanism

**Figure 5:**
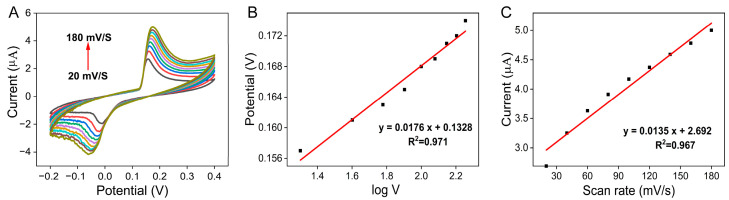
(**A**) Scanning speed test. (**B**) Relationship between scanning speed and peak current. (**C**) Logarithm of scanning speed versus peak voltage.

The feasibility of the prepared sensors for the detection of nitrite was investigated by CV assay. Figure 5A shows the CV curves of the Ag-CeO_2_@C sensor in 100 μM NaNO_2_ plus 0.1 M PBS (pH = 6) solution with different scanning speeds. The available Ag-CeO_2_ sensors displayed distinct redox peaks at 0.16 V and −0.064 V, which indicate the effective diffusion of NO_2_^−^ to the surface of the Ag-CeO_2_@C sensor, while the higher electrocatalytic activity of Ag-CeO_2_@C was attributed primarily to the strong metal-supporting interaction between the Ag nanomaterials and the CeO_2_ substrate, which reduces the aggregation. In addition, the Ag-CeO_2_ nanomaterials provided abundant active sites for nitrous acid. Based on these differences in electrochemical behavior, it is reasonable to believe that Ag-CeO_2_@C has the potential to sense NO_2_.

The effect of the scan rate on NO_2_^−^ oxidation was also studied. Figure 5A shows the CV curves of the Ag-CeO_2_@C sensor with a scan rate ranging from 20 mV/s to 180 mV/s for 1.00 mM NO_2_^−^. As shown in Figure 5B, the oxidation peak current was positively correlated with the increasing scan rate. The linear equation was Ipa (μA) = 0.0135V (mV/s) + 2.692 (R^2^ = 0.967), which demonstrates the adsorption of nitrite and its oxidation products on the electrode surface, implying the oxidation reaction is an adsorption-controlled electrocatalytic process. The peak current value of NO_2_^−^ increased with the increasing scan rate, indicating that the nitrite oxidation reaction is dominated by a typical surface diffusion-controlled process. As shown in Figure 5C, the response of the oxidative peak potential (Ep) and the logarithm of scan rate (logν) was a positive linear relationship: Ep (V) = 0.0176 lg v + 0.1328. The electron transfer coefficient (α) value was calculated to be 0.405 by assuming one electron in the rate-determining step. The electron transfer rate constant (k) of NO_2_^−^ oxidation value was calculated to be 0.54 cm s^−1^.

**Figure 6:**
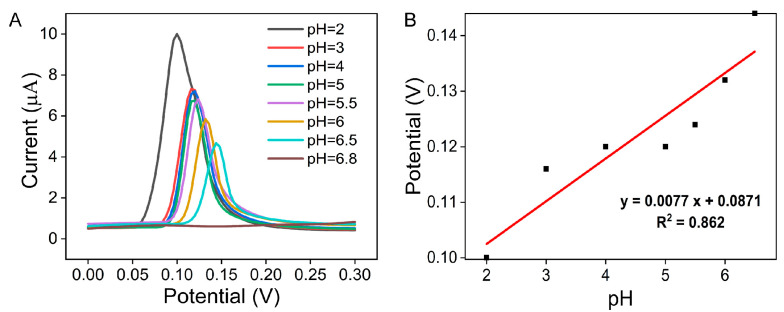
(**A**) DPV test of Ag-CeO_2_@C electrode in 100 μM NaNO_2_ and 0.1 M PBS solutions at different pHs. (**B**) Linear relationship between pH and peak oxidation potential.

To explore the electrooxidative mechanism of NO_2_^−^, the effect of the pH value on the electrochemical response at the Ag-CeO_2_@C sensor was investigated by DPV. Figure 6A shows the DPV curves of Ag-CeO_2_@C in phosphate buffer containing 100 μM NaNO_2_ with different pH values (from 2.0 to 6.8). With the pH value increasing from 2.0 to 6.5, the oxidative peak current decreased, and the peak potential shifted left. With the increase in the pH value from 2.0 to 6.5, a linear relationship between the oxidation peak potential (Epa) and pH was expressed: Epa = 0.0081pH + 0.262 (R^2^ = 0.917), as shown in in Figure 6B. The slope value of 8.1 mV/pH was far from the the theoretical value of 59 mV/pH at 25 ℃, demonstrating that the number of electrons and protons taking part in the electrochemical reaction differed. It was deduced that Ag-CeO_2_@C caused the enhancement of the diffusion, which was favorable for the nitrite diffusion. The CPE in the sensor provides more adsorption sites with rich porous structures. According to the previous work [15,36,37,38,39], we deduced that the single-electron rather than the single-proton mechanism of NO_2_^−^ oxidation at Ag-CeO_2_@C was the following: Under the prerequisite of pH < 6.8, Ag-CeO_2_ is reduced, which improves the electron transfer, and NO_2_^−^ is oxidized to NO_3_^−^. Nitrite ions can combine with protons to form nitrite (1); meanwhile, nitrite accepts protons to generate nitrate and nitric oxide (2) [15]. In addition, Ag nanoparticles play a role in transferring electrons in the reaction [30]. More importantly, NO_2_^−^ was easily absorbed at the surface of the Ag-CeO_2_@C electrode (3). Then, NO_2_ intermediates were generated (4) [36,37]. After that, NO_2_^−^ was converted to NO_3_^−^ with H_2_O under the disproportionated ratio (5, 6) [38,39]. When pH > 6.8, nitrite is difficult to acidically oxidize due to the lack of protons.

(1) $${{\mathrm{NO}}_{2}}^{-}+H^{+}↔{\mathrm{HNO}}_{2}$$

(2) $${{3\mathrm{NO}}_{2}}^{-}+{2H}^{+}↔H_{2}O+{{\mathrm{NO}}_{3}}^{-}+2\mathrm{NO}$$

(3) $${\mathrm{Ag}-\mathrm{CeO}}_{2}@C+{{\mathrm{NO}}_{2}}^{-}↔{\mathrm{Ag}-\mathrm{CeO}}_{2}@C•{{\mathrm{NO}}_{2}}^{-}$$

(4) $$\mathrm{Ag}-{\mathrm{CeO}}_{2}•{{\mathrm{NO}}_{2}}^{-}↔\mathrm{Ag}-{\mathrm{CeO}}_{2}+{\mathrm{NO}}_{2}+e^{-}$$

(5) $${2\mathrm{NO}}_{2}+H_{2}O↔{2H}^{+}+{{\mathrm{NO}}_{2}}^{-}+{{\mathrm{NO}}_{3}}^{-}$$

(6) $${{\mathrm{NO}}_{2}}^{-}+H_{2}O↔{2H}^{+}+{{\mathrm{NO}}_{3}}^{-}+{2e}^{-}$$

### 2.4. Electrochemical Determination of Nitrite

**Figure 7:**
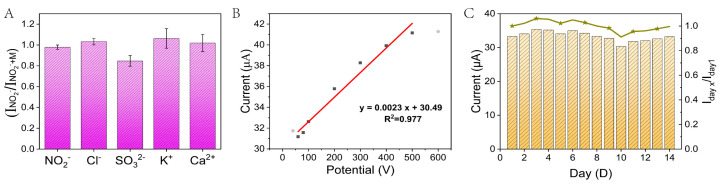
(**A**) Ag-CeO_2_@C interference test at 0.1 M PBS, 100 μM NO_2_^−^, and 50 times the concentration of other ions. (**B**) Linear relationship between NO_2_^−^ concentration and peak oxidation current. (**C**) Fourteen days of continuous monitoring (bar graph shows the current intensity and broken line shows the ratio of the current intensity).

The selectivity is very important for NO_2_^−^ detection based on a Ag-CeO_2_@C sensor in practical applications in view of some inorganic compounds that may coexist in real samples. Possible interference was produced by spiking Cl^−^, SO_3_^2−^, K^+^, and Ca^2+^ into a 100 μM NO_2_^−^ solution, respectively. As shown in Figure 7A, under optimal conditions, there were small changes in the response currents of the NO_2_^−^ peak, although the concentration of NO_2_^−^ (100 μM) was only 1/50 of interference (5000 μM), demonstrating Ag-CeO_2_@C sensor has an excellent anti-interference ability and selectivity.

To achieve the quantitative analysis of NO_2_^−^, a series of concentrations of NO_2_^−^ solutions were determined. The electrochemical analysis of NO_2_^−^ (20.0 μM–800.0 μM) was conducted on the developed Ag-CeO_2_@C system using DPV measurement. The response current signal of the NO_2_^−^ peak was further utilized to study the linear analysis. As shown in Figure 7B, there was an excellent linear correlation of Ip (μA) = 0.0023C (μM) + 30.49 in the range from 40.0 μM to 500.0 μM with a linear correlation coefficient (R^2^ = 0.977). The detection limit was as low as 4.3 μM (7). Three successive quantitative analyses of NO_2_^−^ have good reproducibility with relative standard deviations (RSDs) between 0.89% and 4.39% (*n* = 3). The stability of the Ag-CeO_2_@C sensor was further studied by detecting NO_2_^−^ by continuous measurement for 14 days with an interval of 24 h. As shown in Figure 7C, the response signal of the sensor was still maintained at 91.1% of the initial signal for 14 days (Cyan curve in Figure 7C), which implied that the sensor has good reproducibility and stability, especially in the long term. In addition, the LOD was calculated as follows.

(7) $$\mathrm{LOD}=(\frac{3\times \mathrm{SD}}{\mathrm{Slope}})$$

where SD is the standard deviation of the background noise. Slope is the slope between the signal and the concentration.

### 2.5. Real Sample Analysis

For the application in real samples, the feasibility of the constructed Ag-CeO_2_ sensor was evaluated by determining the amount of NO_2_^−^ in real samples. Three different concentrations of NO_2_^−^ (200, 300, and 400 μM) were respectively spiked into pure milk, mineral water and juice, and chrysanthemum tea samples by the standard addition method and were determined by the developed method in Table 1. The recoveries of NO_2_^−^ in the real samples ranged from 92.1% to 108.1% with RSDs between 0.49% and 9.31% (*n* = 3). High recoveries with allowable RSDs indicated that the prepared sensor and the developed method can be used to determine the amount of nitrite in food testing and water quality monitoring. Thus, the proposal enabled the analysis of NO_2_^−^ with simplicity, convenience, and quickness, which offers potential for onsite analysis.

## 3. Materials and Methods

### 3.1. Chemicals

NaNO_2_, NaOH, Ce(NO₃)₃, AgNO_3_, and ethanol were purchased from Sinopharm Chemical Reagent Co., Ltd. (Shanghai, China). NaNO₃, Na_2_CO_3_, Na_2_SO_4_, KCl, NaF, Na₃PO₄, NaH_2_BO_3_, NaH₂PO₄, and Na₂HPO₄ were purchased from Beijing Chemical Factory, and K_3_Fe(CN)_6_ was purchased from Shanghai Tuhe Industrial Company Limited (Shanghai, China). The 0.1 M phosphate buffer solution (PBS) was adjusted with 0.1 M HCl or NaOH solution.

### 3.2. Preparation and Characterization of Ag-CeO_2_

The preparation method of Ag-CeO_2_ was as described in a previous work [26,30]. In brief, the Ce(NO₃)₃ solution (100 mg/mL, pH = 12) was added to ammonium hydroxide (25 wt%). The precipitate was centrifuged at 8000 r/min for 4 min to make the supernatant neutral. After calcining in a muffle furnace at 500 °C for 5 h, CeO_2_ was achieved. Next, 1 g CeO_2_ was added to 8 mL of the AgNO_3_ solution (0.01 M) and stirred for 5 h. The precipitate was then dried in an oven at 60 °C for 16 h. The dried precipitate was calcined at 500 °C for 3 h. Finally, Ag-CeO_2_ was obtained after washing with acetone, ethanol, and ultrapure water.

The structure and surface morphology of Ag-CeO_2_ were characterized with a scanning electron microscope (SEM), high-resolution transmission electron microscopy (HR-TEM) image (Thermo Fisher Scientific, Shanghai, China), and energy dispersive X-ray (EDX) images (Thermo Fisher Scientific, East Grinstead Town, England). The CeO_2_ material was measured using a Raman spectrometer (Renishow, In Via, Wotton-under-Edge, UK), The crystalline structure of Ag-CeO_2_ was observed with X-ray diffraction spectra (XRD, Rigaku, Smart Lab, Akishima, Japan).

### 3.3. Fabrication of the Modified Electrode

The working electrode (WE) for nitrite detection was fabricated using the Ag-CeO_2_@C modified glassy carbon electrode (GCE). Initially, GCE was polished with alumina powder and then ultrasonically cleaned with distilled water. Ag-CeO_2_ (0.1 g) was mixed with 1 g conductive carbon paste. After stirring well, the Ag-CeO_2_@C slurry was coated onto the pretreated GCE and dried at 70 ℃ for 30 min. The functional Ag-CeO_2_@C WE was obtained. Following the same method, single conductive carbon slurry mixed with CeO_2_ conductive carbon slurry coatings were coated on GCE to obtain CPE WE and CeO_2_@C WE, respectively. In addition, in order to investigate the effect of the conductive carbon on the reaction, we also mixed Ag-CeO_2_ with acrylic acid and coated it on GCE to obtain a Ag-CeO_2_ electrode without conductive carbon paste. The above four electrodes were used as working electrodes for electrochemical detection.

The electrochemical performance including cyclic voltammetry (CV), differential pulse voltammetry (DPV), and electrochemical impedance spectroscopy (EIS) were executed on a CHI-660e electrochemical workstation (CHI 660e Instruments, Shanghai, China, Bio-Sensor Testing Platform) at room temperature. A full three-electrode system was constructed in Figure 1. Ag-CeO_2_@C@GCE was the working electrode. Ag/AgCl was used as a reference electrode (RE), and a Pt wire was a counter electrode (CE). The nitrite sensor made by these three electrodes could be used to detect the nitrite content in drinking water, beverages, and aquaculture water. The photograph of the device was shown in Figure 7. All experiments were carried out in this designed electrochemical sensor.

### 3.4. Real Samples Analysis by Electrochemical Measurements

All the electrochemical experiments were performed on a CHi 660e workstation (Chenhua Instrument Co., Ltd., Shanghai, China). Before detection, the beverage samples were treated based on National Standard for Food Safety Beverages (GB 7101-2022) [40,41,42,43,44]. CV and measurements were carried out at room temperature. The supporting electrolyte was 0.1 M phosphate buffer with Ph = 6. The electrochemical performance of the sensor was tested for CV with potentials ranging from −0.4 V to 0.8 V. The potential range was −0.2 V~0.4 V during the DPV measurement for NaNO_2_.

## 4. Conclusions

In summary, a novel electrochemical sensor was developed for high-efficient and onsite analysis of nitrite. The prepared Ag-CeO_2_ nanoparticles were used to construct a nitrite electrochemical sensor for high electrocatalysis activity, excellent conductivity, and stability. The developed NO_2_^−^ electrochemical sensor showed excellent performance with a linear detection range from 40 μM to 500 μM, and the LOD was reduced to 4.3 μM. More importantly, the established sensor had good reproducibility, selectivity, stability, and strong anti-interference ability. Finally, the Ag-CeO_2_@C sensor was applied to detect nitrite in aquaculture water and real sample beverages. Consequently, we anticipate that this simple, sensitive, and portable sensor will serve as a promising onsite and real-time nitrite detection approach to food testing and environmental monitoring.

## Figures and Tables

**Table 1:** The recovery of nitrite spiked into real samples (*n* = 3).

Real Samples	Spiked Nitrite (μM)	Detection Nitrite (μM)	Recovery (%)	RSD (%, *n* = 3)
MengNiu Milk	200	195.2	97.6	0.98
	300	293.4	97.8	1.33
	400	382.4	95.6	2.92
ChangFu Milk	200	184.1	92.1	1.81
	300	311.6	103.9	9.31
	400	407.0	101.7	0.85
Fruit Juice	200	205.7	102.8	1.17
	300	328.2	101.7	0.49
	400	368.9	92.2	3.27
Chrysanthemum Tea	200	207.4	103.7	2.26
	300	295.2	98.4	0.49
	400	403.1	100.8	4.19
Evergrands Spring	200	129.7	96.4	1.17
	300	299.6	99.8	3.11
	400	392.2	98.1	2.17
Aquaculture water	200	216.2	108.1	8.97
	300	282.4	94.1	3.83
	400	424.2	106.2	2.14

## Data Availability

Data are contained within the article.
